# Quality dentistry and ethical dental practice

**DOI:** 10.1002/cre2.128

**Published:** 2018-08-30

**Authors:** Asbjorn Jokstad

The annual meeting of the International Association of Dental Research (IADR) ended last week in London, U.K. It can be stated once again that these annual meetings appear to be the most authoritative venue to learn about current cutting ‐edge science in oral and craniofacial health and medicine. Peer‐selected basic and clinical research is presented in different ways, including short oral presentations, posters, hands‐on courses, topical symposia, lunch‐and‐learn sessions and keynote lectures. As a clinician and investigator I appreciate the symposia termed clinical track because the focus is on new scientific findings relevant for our patients, reasons for implementing new findings into daily patient practice, and anticipated values for both clinicians and patients. Yet, one symposium this year titled *Quality in dentistry* made me stop and reflect. Two young investigators did an excellent presentation of their systematic review on *quality systems in dentistry,* but I was somewhat disturbed to recognize that they presented various schemes for scoring patient satisfaction. Worse, the rest of the symposium session proceeded without any of the other presenters mentioning core terms such as patient expectations, patient rights, practice of informed consent and patient autonomy. My gut feeling was that the topic was highly pertinent, but without an involvement of any experienced reflective clinician, it is so easy to fall into an us‐versus‐them thinking, perhaps even with a flair of elitist ivory‐tower perspective on what wet‐finger‐dentists ought to do.

I admit that my contributions on the topic of *Quality in dentistry* have not provided clear answers to many issues in real‐world clinical dentistry (Jokstad A., 2008; Jokstad A., Braegger U., Brunski J.B., Carr A.B., Naert I., Wennerberg A., 2003: Jokstad A., Bayne S., Blunck U., Tyas M., Wilson N.H., 2001; Söderholm K.J., Tyas M., Jokstad A., 1998; Jokstad A. & Mjör I.A., 1989). However, to my defense I have practiced as a clinical dentist for near four decades and in spite of a continuous focus on evidence‐based care and ethical practice combined with perceived high patient satisfaction, there may be unforeseen pitfalls. Societal conditions, and perhaps ill fate, can confound and cause chagrin. The following sad story contains aspects that I find difficult to reconcile as a scientist, as an ethically cognizant researcher, as a reflective practitioner and as a professional colleague. Although the venue of this particular story is in rural Norway, I am sure that similar situations arise elsewhere. I urge clinicians who read this editorial to reflect on how they would act if they somehow were caught in a scenario described below.

A young graduate established a private practice in the early sixties in a township in rural Norway. The caries rate at the time was extremely high, and he was inundated with patients since the next dentist was miles away. All cherished him because he provided acute care practically 24/7. After about 50 years of dedicated service, the local newspaper honored him with an interview and photograph where it was also informed that his practice had been taken over by a younger colleague. His patient portfolio at the time exceeded 3000, mostly local, individuals. The new dentist rapidly identified extensive untreated pathology in a few patients, as well as multiple technically inadequate endodontic and coronal restorations. Documentation was sent to the Norwegian Agency for patient injury compensation (NPE), where the undersigned is a contracted expert in clinical dentistry. Initially, I had considerable doubts for various reasons to state that the patients had received inferior care, but in the end, we endorsed the professional judgment of the new dentist, and the patients were compensation for poor oral health care. The outcome prompted an outcry amongst the very senior township citizens, and rumors resulted in further applications for injury compensation, which were also approved. As expected, the professional legacy of the retired dentist was a disaster within just a few months. Some would say the retired dentist was a victim and the new dentist extremely un‐collegial. Others would say that the real victims were the patients due to supervised neglect over many years and that the new dentist was very brave when he proceeded to question the care provided by the local township hero.

Yet, this issue is not so white‐black. At the time of his retirement, 3000 individuals attended the clinic for reasons we don't know. We can, however, expect that both patient expectations were addressed, and that patient satisfaction was high, but can these criteria really be considered as a measure of quality care? In theory, the dentist could have informed say, two thousand of his patients to find another dentist since he would need to work more downstream than upstream. He would have obtained more time for fewer patients, but simultaneously consigned many of his township neighbors to travel to another rural township for dental care, so the suggestion would likely not be popular. Moreover, a private practice built on nepotism in a rural township is also likely non‐sustainable. He could also had, in theory, advised his patients that because his time was pressed, he would not be able to undertake annual recalls with clinical and radiological examinations, nor provided advanced periodontal treatments. Would this strategy constitute lack of quality dental care and ethical practice? Moreover, if your waiting room daily is crowded with patients with signs and symptoms, is it really a practice of best ALARA principle to add to the radiographic examinations a couple of bite‐wings for the records? In sum, how can one establish treatment equity amongst patients when resources are limited in a rural township in Scandinavia or elsewhere? Is this a question that can be answered scientifically? One should perhaps be a bit humble and not sit on a high horse in 2018 and state that the dentist that was singled out in this narrative ought to have done this and what not in the five previous decades.

Even so, most countries today have legislated patient's bill of rights, which trump eventual considerations that single medical or dental practitioners may have established. These include, amongst other elements, a right to obtain information about their health condition and autonomy over treatment decisions including consequences of not undertaking any interventions. Any third‐party assessor who will judge whether a patient has received care according to a bill of rights will scrutinize patient charts for this documentation. Hence, dear colleagues who still complete hand‐written notes without recorded signs or symptoms, tentative or definite diagnoses, alternative treatment options and patient opinions, consider what your professional legacy will be when a new dentist in the foreseeable future will manage your current patients. Quality dentistry and ethical dental practice goes beyond assessing patient satisfaction.
